# Extracting Information about the Rotator Cuff from Magnetic Resonance Images Using Deterministic and Random Techniques

**DOI:** 10.1155/2015/974562

**Published:** 2015-01-12

**Authors:** F. A. De Los Ríos, M. Paluszny

**Affiliations:** ^1^Universidad Nacional de Colombia, Sede Bogotá, Bogotá 11001, Colombia; ^2^Universidad Nacional de Colombia, Sede Medellín, Medellín 05001, Colombia

## Abstract

We consider some methods to extract information about the rotator cuff based on magnetic resonance images; the study aims to define an alternative method of display that might facilitate the detection of partial tears in the supraspinatus tendon. Specifically, we are going to use families of ellipsoidal triangular patches to cover the humerus head near the affected area. These patches are going to be textured and displayed with the information of the magnetic resonance images using the trilinear interpolation technique. For the generation of points to texture each patch, we propose a new method that guarantees the uniform distribution of its points using a random statistical method. Its computational cost, defined as the average computing time to generate a fixed number of points, is significantly lower as compared with deterministic and other standard statistical techniques.

## 1. Introduction and Preliminaries

The rotator cuff is a group of muscles and tendons connected to the humerus head whose function is the mobility and stability of the shoulder. The anatomy and physiology of the rotator cuff is complex and it is interconnected to other muscle groups in the shoulder. These muscle groups perform multiple functions and are often stressed during various activities. Disease of the rotator cuff is the most common cause of shoulder pain and dysfunction in adults; see [1, 2].

A rotator cuff tear is a tear of one or more of the tendons of the four rotator cuff muscles; these can be classified according to their depth into full thickness and partial thickness tears; see [1, 2]. Magnetic resonance imaging plays a major role in helping to identify rotator cuff affections. In fact many surgeons rely on magnetic resonance images to assist in decision making and presurgical planning for patients with rotator cuff pain; see [3].

In this research, we explore methodologies to extract information about the rotator cuff from magnetic resonance images; particularly we emphasize on the detection of supraspinatus tendon partial tears. We consider special domains on ellipsoids and in particular triangular Bézier patches to cover the humerus head close to the affected area; these patches are textured with the information of the magnetic resonance images using the trilinear interpolation technique; see [4]. To texture the patch two techniques are considered: a deterministic method and a random method. The random generation methodology can be chosen in a way that it ensures a uniform distribution of points and hence it avoids the systematic accumulation in specific areas. Therefore, we define families of special ellipsoidal patches, and we present an algorithm for the random generation of points uniformly distributed on the patch (see [5]). The computational efficiency of this algorithm, measured as the average computing time to generate a fixed number of points, is compared with other nonrandom generating techniques, namely, the classical de Casteljau algorithm and the Bernstein polynomial evaluation of triangular Bézier patches; see [6–11].

The paper is organized as follows. In Section 2, we describe a statistical technique to generate uniformly distributed points on patches, focusing on the case of the upper half of an ellipsoid. In Section 3, we center our discussion on the sphere and the ellipsoid of revolution, and we compare them with the case of the general ellipsoid. Finally, in Section 5 we present the comparison of the computational costs.

## 2. Random Evaluation of Uniformly Distributed Points

In this section, we review the problem of sampling uniformly distributed points on a parametric surface that lies on the upper half of an ellipsoid centered at the origin with semiaxes *a*_1_ ≥ *a*_2_ ≥ *a*_3_ > 0. Let us denote this surface by *s*(**x**) = *s*(*x*_1_, *x*_2_) = (*x*_1_, *x*_2_, *x*_3_(*x*_1_, *x*_2_)). Namely, *s* is defined as the graph of the function *x*_3_ on the domain *D*:

(1) x3=a31−x1a12−x2a22,D=x:α2a21−x1a12<x2<α1a21−x1a12,  1−x1a12β2<x1<β1,

where *i* = 1,2 and |*α*_*i*_| ≤ 1, |*β*_*i*_| ≤ *a*_1_. We want to generate uniformly distributed points in the image of the domain *D*. This allows us to guarantee that the generated points will not accumulate; this feature is very important for the visualization.

That is to say, we will generate values of a random vector *X* = (*X*_1_, *X*_2_) such that *s*(*X*) is uniformly distributed on *s*(*D*). This condition means that, given *A* ∈ *R* ^2^ in *D* and denoting by *λ* the Lebesgue measure of dimension 2 for the surface, the probability of *s*(*X*) ∈ *s*(*A*) must be equal to *λ*(*s*(*A*))/*λ*(*s*(*D*)); see [5, 12]. Taking into account the fact that *x*_3_ is continuously differentiable, according to [13] the uniformity implies that

(2) Pω:sXω∈sA =Pω:Xω∈A =λsAλsD =∫A1+∂x3/∂x12+∂x3/∂x22dxλsD.

The expression (2) implies that *X* must have density with respect to the Lebesgue measure of dimension 2, given by

(3) $$\begin{array}{c} f\left(x_{1},x_{2}\right)=\left(\frac{1}{\lambda \left(s\left(D\right)\right)}\right)\sqrt{1+{\left(\frac{\partial x_{3}}{\partial x_{1}}\right)}^{2}+{\left(\frac{\partial x_{3}}{\partial x_{2}}\right)}^{2}}, \end{array}$$

if **x** ∈ *D* and 0 otherwise.

In this sense, if we compute the partial derivatives of *x*_3_ with respect to *x*_1_ and *x*_2_ and let *δ*_1_ = 1 − (*a*_3_/*a*_1_)^2^, *δ*_2_ = 1 − (*a*_3_/*a*_2_)^2^, and *κ* = (*λ*(*s*(*D*)))^−1^, the density function *f* for **x** ∈ *D* can be written as

(4) $$\begin{array}{c} f\left(x_{1},x_{2}\right)=\kappa \sqrt{\frac{1-\delta_{1}{\left(x_{1}/a_{1}\right)}^{2}-\delta_{2}{\left(x_{2}/a_{2}\right)}^{2}}{1-{\left(x_{1}/a_{1}\right)}^{2}-{\left(x_{2}/a_{2}\right)}^{2}}}, \end{array}$$

and, given that *a*_1_ ≥ *a*_2_ ≥ *a*_3_ > 0, we have 1 > *δ*_1_ ≥ *δ*_2_ ≥ 0. Namely, to satisfy the uniformity condition, it is necessary to generate the random vector *X* with distribution *f* given by (4).

The strategy to generate *X* consists in making a change of variables to transform *D* into a rectangle. Let **y** = (*y*_1_, *y*_2_) = *g*(*x*_1_, *x*_2_) be a variable change such that its inverse (*x*_1_, *x*_2_) = *g* ^−1^(*y*_1_, *y*_2_) is

(5) $$\begin{array}{c} x_{1}=a_{1}y_{1},\;\;x_{2}=a_{2}y_{2}\sqrt{1-y_{1}^{2}},\;\text{in}\;\text{the}\;\text{domain}\\ D_{g}=\left\{\left(y_{1},y_{2}\right):\frac{\beta_{2}}{a_{1}}<y_{1}<\frac{\beta_{1}}{a_{1}},\;\;\alpha_{2}<y_{2}<\alpha_{1}\right\}. \end{array}$$

The mapping *g* is one-to-one and continuously differentiable so, using the change of variables theorem, we find that the density of the vector *Y* = (*Y*_1_, *Y*_2_) = *g*(*X*) with respect to the Lebesgue measure is

(6) $$\begin{array}{c} f_{g}\left(y\right)=\kappa a_{1}a_{2}\sqrt{1-\delta_{1}y_{1}^{2}}\sqrt{\frac{\left(1-m\left(y_{1}\right)y_{2}^{2}\right)}{\left(1-y_{2}^{2}\right)}} \end{array}$$

for **y** ∈ *D*_*g*_, and *m*(*y*_1_) = *δ*_2_(1 − *y*_1_^2^)/(1 − *δ*_1_ *y*_1_^2^); see [14].

Note that the problem of generating *X* can be solved by generating the random vector *Y* and then pulling back by *g*.

Next, to generate the random vector *Y* for a spherical patch or for a patch of an ellipsoid of revolution, we will consider a simplified technique. This is what we will do in the next section and we will compare it with the general ellipsoid.

## 3. Patch Evaluation: Sphere and Ellipsoid

### 3.1. Sphere

In the case of a sphere we have *a*_1_ = *a*_2_ = *a*_3_ and *δ*_1_ = *δ*_2_ = 0; hence the joint density of *Y* on *D*_*g*_ is

(7) $$\begin{array}{c} f_{g}\left(y\right)=\kappa a_{1}^{2}\sqrt{\frac{1}{\left(1-y_{2}^{2}\right)}}. \end{array}$$

In this case the function *f*_*g*_(**y**) can be written as the product of its two marginal functions *f*_*g*,1_ and *f*_*g*,2_; therefore the random variables *Y*_1_ and *Y*_2_ are independent.

The independence condition allows for the generation of *Y* using the inverse transform method; see [5]. We consider inverse images of the accumulated distributions of *f*_*g*,1_ and *f*_*g*,2_, namely, *F*_*g*,1_^−1^ and *F*_*g*,2_^−1^, respectively. To generate *Y*, we take into account the fact that if *U* = (*U*_1_, *U*_2_) are uniformly distributed in the interval (0,1), then *Y* = (*F*_*g*,1_^−1^(*U*_1_), *F*_*g*,2_^−1^(*U*_2_)) is distributed with density *f*_*g*_. Explicitly, we compute

(8) $$\begin{array}{c} F_{g,1}^{-1}\left(U_{1}\right)=\frac{\left(\beta_{1}U_{1}+\beta_{2}\left(1-U_{1}\right)\right)}{a_{1}},\\ F_{g,2}^{-1}\left(U_{2}\right)=\sin\left(U_{2}\arcsin\left(\alpha_{1}\right)+\left(1-U_{2}\right)\arcsin\left(\alpha_{2}\right)\right). \end{array}$$

### 3.2. Ellipsoid of Revolution

For an ellipsoid of revolution we have *a*_2_ = *a*_3_ and *δ*_2_ = 0; hence the joint density of *Y* on *D*_*g*_ is

(9) $$\begin{array}{c} f_{g}\left(y\right)=\kappa a_{1}a_{2}\sqrt{1-\delta_{1}y_{1}^{2}}\sqrt{\frac{1}{\left(1-y_{2}^{2}\right)}}. \end{array}$$

Let us note that the random variables *Y*_1_, *Y*_2_ whose marginal distributions we denote by *f*_*g*,1_ and *f*_*g*,2_, respectively, are statistically independent; therefore it is again possible to use the inverse transform method to generate the random vector *Y*.

Namely, to generate *Y*, we generate the vector *U* = (*U*_1_, *U*_2_) of independent components and uniformly distributed and then we compute the vector *Y* = (*F*_*g*,1_^−1^(*U*_1_), *F*_*g*,2_^−1^(*U*_2_)). Specifically,

(10) $$\begin{array}{c} F_{g,1}^{-1}\left(U_{1}\right)=\phi^{-1}\left(U_{1}\phi \left(\frac{\beta_{1}}{a_{1}};\delta_{1}\right)+\left(1-U_{1}\right)\phi \left(\frac{\beta_{2}}{a_{1}};\delta_{1}\right);\delta_{1}\right),\\ F_{g,2}^{-1}\left(U_{2}\right)=\sin\left(U_{2}\arcsin\left(\alpha_{1}\right)+\left(1-U_{2}\right)\arcsin\left(\alpha_{2}\right)\right), \end{array}$$

where

(11) ϕt;δ1=∫1−δ1t2dt=2δ1−1δ1t1−δ1t2+arcsinδ1t.

The function *ϕ* is strictly monotone on the interval [*β*_2_/*a*_1_, *β*_1_/*a*_1_], so its inverse function *ϕ* ^−1^ exists, and it is also strictly monotone.

The function *ϕ* ^−1^ has no closed analytic expression but it can be approximated numerically. We would like the approximation to be independent of the shape parameter *δ*_1_. Consider the random variable $Z=\sqrt{\delta_{1}}Y_{1}$. It has accumulated distribution:

(12) $$\begin{array}{c} F_{Z}\left(z\right)=\frac{\left(\phi \left(z;1\right)-\phi \left(r_{2};1\right)\right)}{\left(\phi \left(r_{1};1\right)-\phi \left(r_{2};1\right)\right)}, \end{array}$$

and it is defined on the interval (*r*_2_, *r*_1_), where $r_{i}=\left(\beta_{i}/a_{1}\right)\sqrt{\delta_{1}}$. Let *F*_*Z*_∗__ be the distribution *F*_*Z*_ in the special case that the interval (*r*_2_, *r*_1_) is (−1,1). In the literature *F*_*Z*_∗__ is referred to as the semicircular distribution; see [15]. In accordance with [16], it is possible to sample *Z* from the inverse function *F*_*Z*_∗__^−1^(*u*):

(13) FZ−1u=FZ∗−1uFZ∗r1+1−uFZ∗r2=ϕ−1uϕr1;1+1−uϕr2;1;1,

for *u* ∈ (0,1) and *r*_*i*_ as above. In this sense, to generate *Z*, we could draw a random number *U* with uniform density on the interval *I* = (*ϕ*(*r*_2_; 1), *ϕ*(*r*_1_; 1)) and then evaluate *ϕ* ^−1^(*U*; 1).

#### 3.2.1. Approximation of *ϕ* ^−1^

To generate the random variable *Z*, we approximate the function *ϕ* ^−1^(*u*; 1) with a cubic spline. More precisely, let *s*_0_ < *s*_1_ < ⋯<*s*_*n*−1_ be a partition of *I*, we compute *ϕ*(*s*_*k*_; 1) for *k* = 0,1,…, *n* − 1, and we do monotone piecewise cubic Hermite interpolation of the data set {*ϕ*(*s*_*k*_; 1), *s*_*k*_}_*k*=0_^*n*−1^ according to the method described in [17]. There, the authors derive the necessary and sufficient conditions for a cubic Hermite interpolator to be piecewise monotone and develop an algorithm to build it for a monotone data set. In MATLAB, for example, we can do monotone interpolation with the function pchip.

For the sake of completeness, next we review two general methods to sample random variables with arbitrary distributions. We will compare these with the special techniques presented here for the ellipsoid of revolution.

#### 3.2.2. Acceptance-Rejection Method

The acceptance-rejection method is one of the most useful general methods for sampling from general distributions. It can be applied to both discrete and continuous distributions and even to multidimensional distributions although its efficiency rapidly decreases as the dimension increases [5].

We want to simulate a random variable *X* with density given by *f*(*x*). Let us suppose that we have a method to sample a random variable *Y* with density *g*(*y*). In addition, let us assume that it is possible to bound the ratio *f*(*x*)/*g*(*x*) by a constant *c* > 0; *c* = sup⁡_*x*_{*f*(*x*)/*g*(*x*)}.

The acceptance-rejection method is based on the following algorithm [5].

1. Generate *X* with density *g*(*x*).
2. Generate *U* with uniform density on the interval (0,1).
3. If *U* ≤ *f*(*x*)/(*cg*(*x*)) output *X*; else return to step (2).

This is to say, generate *X* with density *g*(*x*) and accept it with probability *f*(*x*)/(*cg*(*x*)); else reject *X* and try again. The efficiency of the acceptance-rejection method is defined as *P*(*U* ≤ *f*(*X*)/(*cg*(*X*))), and it is 1/*c*; see [5].

Specifically, to simulate the random variable *Y*_1_ whose density we have denoted by *f*_*g*,1_(*y*_1_), we could use the acceptance-rejection algorithm taking *f*(*x*) = *f*_*g*,1_(*x*) and *g*(*x*) = (*a*_1_/(*β*_1_ − *β*_2_))*χ*_{(*β*_2_/*a*_1_, *β*_1_/*a*_1_)}_(*x*). Namely, the random variable *X* will be sampled uniformly on the interval (*β*_2_/*a*_1_, *β*_1_/*a*_1_).

To determine the constant *c* in the algorithm, let us note that the maximum of the function *f*_*g*,1_(*x*) which will be denoted by *f* ^*^ is *f*_*g*,1_(0) if 0 ∈ (*β*_2_/*a*_1_, *β*_1_/*a*_1_) or max⁡(*f*_*g*,1_(*β*_2_/*a*_1_), *f*_*g*,1_(*β*_1_/*a*_1_)) otherwise. Choosing *c* = ((*β*_1_ − *β*_2_)/*a*_1_)*f* ^*^ and taking into account the fact that *g*(*x*) = (*a*_1_/(*β*_1_ − *β*_2_)) on the interval (*β*_2_/*a*_1_, *β*_1_/*a*_1_), it is clear that *f*(*x*) ≤ *cg*(*x*).

#### 3.2.3. Hastings Algorithm

The Hastings algorithm is a popular technique to simulate random variables with densities difficult to handle. Given a particular probability density *π*(**x**) defined on a region *S* and an arbitrary probability density *q*(**x**∣**y**) that depends on (**x**, **y**) ∈ *S* × *S*, a sketch of Hastings' algorithm (see [18]) is as follows.

(1) Select an initial point *X*_0_ ∈ *S*.
(2) On the *t* iteration do the following.
  (a) Generate *X*′ of the density *q*(**x**∣**x**_**t**−1_).
  (b) Compute
    (14) $$\begin{array}{c} \rho \left(X_{t-1},X^{\prime }\right)=\min\left\{1,\frac{\pi \left(X^{\prime }\right)q\left(X_{t-1}\;|\;X^{\prime }\right)}{\pi \left(X_{t-1}\right)q\left(X^{\prime }\;|\;X_{t-1}\right)}\right\}. \end{array}$$
  (c) Take
    (15) $$\begin{array}{c} X_{t}=\left\{\begin{array}{cc} X^{\prime } & \text{with}\;\text{probability}\;\rho \left(X_{t-1},X^{\prime }\right)\\ X_{t-1} & \text{with}\;\text{probability}\;1-\rho \left(X_{t-1},X^{\prime }\right). \end{array}\right. \end{array}$$
(3) Repeat step (2) until equilibrium is reached.

In other words, in the iteration *t*, the Hastings sampler chooses a sample *X*′ of the instrumental density *q*; this density could depend on the last sampled variable *X*_*t*−1_. The algorithm decides to accept the new random variable *X*′ according to (15).

The Hastings algorithm induces a Markov chain whose stationary distribution has density *π*(**x**), regardless of the choice of the instrumental density *q*. In practice, *q* is chosen as being easy to sample and close to the density *π*(**x**).

### 3.3. General Ellipsoid

We can apply Hastings or acceptance-rejection method to each of the components of a vector but this leads to the computation of elliptic integrals which is computationally very expensive.

Alternatively using Hastings on the random vector is less costly but exceeds by a factor much greater the deterministic evaluation using the classical methods of de Casteljau and Bézier.

## 4. Surfaces in Medical Visualization

Medical imaging is the set of techniques and procedures used to create digital images of parts of the human body for clinical purposes or medical research. Digital images are composed of individual pixels, to which discrete brightness or color values are assigned. They can be efficiently processed, objectively evaluated, and made available at many places at the same time by means of appropriate communication networks and protocols, such as Picture Archiving and Communication Systems (PACS) and the Digital Imaging and Communications in Medicine (DICOM) protocol, respectively.

In fact, DICOM is a standard for handling, storing, printing, and transmitting information in medical imaging; see [19].

Since the discovery of X-rays by Wilhelm Conrad Rötgen in 1895, medical images have become a major component of diagnostics, treatment planning and procedures, and follow-up studies; therein lies its importance in healthcare. Medical images are acquired using a variety of techniques and devices, including conventional radiography, computed tomography (CT), magnetic resonance imaging (MRI), ultrasound, and nuclear medicine. As stated in the preliminaries we will focus on rotator cuff MRI.

The usual technique for the visualization of rotator cuff tears is by inspection of a sequence of parallel sections. We propose an alternative technique. We look at a curved surface along the humerus near the suspected area of the tear. This exhibits the full tear extension in a single view which is positioned in a natural way with respect to the humerus head. We choose the curved surface fitting the humerus head to be a patch of sphere or ellipsoid of revolution. These are estimated using standard least squares techniques.

For the visualization of the tear we need to texturize it with the MRI data, that is, to evaluate (To evaluate the patch means to find the coordinates of all its points.) the patch and assign a gray level to each point.

In the next section we look at various evaluation techniques, deterministic and random, and present comparison tables of their relative central processing unit (CPU) performance.

## 5. Comparison of Various Surface Evaluation Techniques

To generate the points of the patch we have several options. For the sake of comparison we will consider both a sphere octant and an ellipsoid of revolution octant. As mentioned in the introduction the patch evaluation methods are of two types: deterministic and random.

In the case of a patch on the sphere a uniform distribution of points can be generated using the inverse transform method. On the other hand, if the surface is an ellipsoid of revolution we generate its points by combining the method of inverse transform in one dimension with methods such as the method based on the approximation of *ϕ* ^−1^, acceptance and rejection, and Hastings algorithm.

From the deterministic point of view, we will use the representation of the octant as a triangular Bézier patch; see [6].

A triangular Bézier patch of degree *n* with control network **b**_**i**_ ∈ *E* ^3^ and associated weights *w*_**i**_ ∈ *R* is a parametric surface **b**_0_^*n*^(**u**) ∈ *E* ^3^. The parameter **u** = (*u*, *v*, *w*) represents the barycentric coordinates of a point in a triangular patch and the multi-index **i** = (*i*_1_, *i*_2_, *i*_3_) is related with a particular location in a triangular array of degree *n*. The triangular Bézier patch can be evaluated by means of either the de Casteljau algorithm or the Bernstein representation.

The de Casteljau algorithm is a recursive procedure to compute a point on the triangular patch as follows (see [6]):

(16) biru =uwi+e1bi+e1r−1u+vwi+e2bi+e2r−1u+wwi+e3bi+e3r−1uwir,

where **e**_1_, **e**_2_, and **e**_3_ are the unit canonical vectors:

(17) $$\begin{array}{c} w_{i}^{r}\left(u\right)=uw_{i+e_{1}}\left(u\right)+vw_{i+e_{2}}\left(u\right)+ww_{i+e_{3}}\left(u\right),\\ r=1,\ldots ,n;\;\left|i\right|=n-r;\;b_{i}^{0}=b_{i}. \end{array}$$

Then **b**_0_^*n*^(**u**) is the position of the point corresponding to **u**.

The second deterministic method uses homogeneous Bernstein polynomials *B*_**i**_^*n*^ (see [6]):

(18) $$\begin{array}{c} b^{n}\left(u\right)=b_{0}^{n}\left(u\right)=\frac{\sum_{\left|i\right|=n}w_{i}b_{i}B_{i}^{n}\left(u\right)}{\sum_{\left|i\right|=n}w_{i}B_{i}^{n}\left(u\right)}, \end{array}$$

where **b**_**i**_ are the control points and *w*_**i**_ are the weights.

De Casteljau and Bézier patch evaluation are the two standard techniques to compute points of a triangular patch. The Bézier evaluation is given by an analytic formula and the algorithm of de Casteljau is iterative but has some potentially useful side products such as the computation of the surface curvature. Nevertheless, none of these deterministic algorithms guarantees a uniform distribution of points on the patch.

The random technique is based on methodologies that provide asymptotically uniform distribution of points on the patch. In practical terms this means that as the resolution is increased the information is retrieved more faithfully on the whole patch.

Table 1 illustrates the CPU performance of the algorithms in a standard personal computer (PC) for the generation of *n* points on an octant of a sphere (The sphere octant is realized in a rational Bézier patch of degree 4.). Our experiments show that, regardless of the number of points generated, the algorithm based on the inverse transform technique is faster than the deterministic algorithms. Further, the efficiency ratio for the random method with respect to the deterministic methods increases as the number of points generated increases.

For the ellipsoid, the comparison is given in Table 2. Our results suggest that, regardless of the number of points generated, the algorithm based on an approximation of the inverse transform technique is faster than the other algorithms both deterministic and random. In addition, the efficiency ratio for the discussed method with respect to the other methods increases as the number of points generated increases.

## 6. Conclusions

Visualization of medical data along curved surface patches is potentially a useful tool in medical research and clinical praxis. We consider the specific application in the case of the rotator cuff. We propose a family of patches fitted to the humerus head and texturing methods which are highly competitive with the standard Bernstein-de Casteljau deterministic algorithms.

## Supplementary Material

In the video, we can visualize a sequence of spherical surface patches with common center and whose radius is variable in the time. The patches are shaded with information of a particular magnetic resonance of the rotator cuff.

## Figures and Tables

**Table 1:** Average computing time to generate *n* points on the octant of a sphere (seconds).

Method	*n* = 1035	*n* = 10011	*n* = 50086
Inverse transform	0.000168	0.000987	0.003989
Bernstein representation	0.003485	0.026029	0.125881
De Casteljau algorithm	0.002890	0.043633	0.212243

**Table 2:** Average computing time to generate *n* points on the octant of an ellipsoid of revolution (seconds).

Method	*n* = 1035	*n* = 10011	*n* = 50086
ϕ^−1^ approximation	0.001318	0.003352	0.010890
Acceptance-rejection	0.013956	0.125176	0.636031
Hastings	0.406324	2.225163	10.38507
Bernstein representation	0.003517	0.026096	0.126542
De Casteljau algorithm	0.002904	0.044050	0.212913

## References

[1] Opsha O., Malik A., Baltazar R. (2008). MRI of the rotator cuff and internal derangement. *European Journal of Radiology*.

[2] American Academy of Orthopaedic Surgeons (2013). *Rotator Cuff Tears: Surgical Treatment Options*.

[3] Tuite M. J. (2012). Magnetic resonance imaging of rotator cuff disease and external impingement. *Magnetic Resonance Imaging Clinics of North America*.

[4] Rajon D. A., Bolch W. E. (2003). Marching cube algorithm: review and trilinear interpolation adaptation for image-based dosimetric models. *Computerized Medical Imaging and Graphics*.

[5] Kroese D. P., Taimre T., Botev Z. I. (2011). *Handbook of Monte Carlo Methods*.

[6] Farin G. (2002). *Curves and Surfaces for CAGD: A Practical Guide*.

[7] Prautzsch H., Boehm W., Paluszny M. (2002). *Bezier and B-Spline Techniques*.

[8] Boehm W., Müller A. (1999). On de Casteljau's algorithm. *Computer Aided Geometric Design*.

[9] Pottmann H. (1995). Rational curves and surfaces with rational offsets. *Computer Aided Geometric Design*.

[10] Farouki R. T. (2012). The Bernstein polynomial basis: a centennial retrospective. *Computer Aided Geometric Design*.

[11] Farin G., Piper B., Worsey A. J. (1987). The octant of a sphere as a nondegenerate triangular Bézier patch. *Computer Aided Geometric Design*.

[12] Smith R. L. (1984). Efficient Monte Carlo procedures for generating points uniformly distributed over bounded regions. *Operations Research*.

[13] Taylor M. (2006). *Measure Theory and Integration*.

[14] Billingsley P. (1995). *Probability and Measure*.

[15] Hogg R., Craig A., McKean J. (2005). *Introduction to Mathematical Statistics*.

[16] Nadarajah S., Kotz S. (2006). Programs for computing truncated distributions. *Journal of Statistical Software*.

[17] Fritsch F. N., Carlson R. E. (1980). Monotone piecewise cubic interpolation. *SIAM Journal on Numerical Analysis*.

[18] Levine R. A., Yu Z., Hanley W. G., Nitao J. J. (2005). Implementing componentwise Hastings algorithms. *Computational Statistics & Data Analysis*.

[19] Deserno T. M. (2011). *Biomedical Image Processing*.

